# Metal-independent variants of phosphoglycerate mutase promote resistance to nutritional immunity and retention of glycolysis during infection

**DOI:** 10.1371/journal.ppat.1007971

**Published:** 2019-07-25

**Authors:** Jana N. Radin, Jessica L. Kelliher, Paola K. Párraga Solórzano, Kyle P. Grim, Rouhallah Ramezanifard, James M. Slauch, Thomas E. Kehl-Fie

**Affiliations:** 1 Department of Microbiology, University of Illinois at Urbana-Champaign, Urbana, IL, United States of America; 2 Departmento de Ciencias de la Vida, Universidad de las Fuerzas Armadas ESPE, Sangolquí, Ecuador; 3 Carl R. Woese Institute for Genomic Biology, University of Illinois at Urbana-Champaign, Urbana, IL, United States of America; National Institutes of Health, UNITED STATES

## Abstract

The ability of *Staphylococcus aureus* and other pathogens to consume glucose is critical during infection. However, glucose consumption increases the cellular demand for manganese sensitizing *S*. *aureus* to host-imposed manganese starvation. The current investigations were undertaken to elucidate how *S*. *aureus* copes with the need to consume glucose when metal-limited by the host. A critical component of host defense is production of the manganese binding protein calprotectin. *S*. *aureus* has two variants of phosphoglycerate mutase, one of which is manganese-dependent, GpmI, and another that is manganese-independent, GpmA. Leveraging the ability to impose metal starvation in culture utilizing calprotectin revealed that the loss of GpmA, but not GpmI, sensitized *S*. *aureus* to manganese starvation. Metabolite feeding experiments revealed that the growth defect of GpmA when manganese-starved was due to a defect in glycolysis and not gluconeogenesis. Loss of GpmA reduces the ability of *S*. *aureus* to cause invasive disease in wild type mice. However, GpmA was dispensable in calprotectin-deficient mice, which have defects in manganese sequestration, indicating that this isozyme contributes to the ability of *S*. *aureus* to overcome manganese limitation during infection. Cumulatively, these observations suggest that expressing a metal-independent variant enables *S*. *aureus* to consume glucose while mitigating the negative impact that glycolysis has on the cellular demand for manganese. *S*. *aureus* is not the only bacterium that expresses manganese-dependent and -independent variants of phosphoglycerate mutase. Similar results were also observed in culture with *Salmonella enterica* serovar Typhimurium mutants lacking the metal-independent isozyme. These similar observations in both Gram-positive and Gram-negative pathogens suggest that expression of metal-independent glycolytic isozymes is a common strategy employed by bacteria to survive in metal-limited environments, such as the host.

## Author summary

Pathogens, such as *Staphylococcus aureus* and *Salmonella* species, must be able to consume glucose in order to cause infection. However, glycolysis can increase the need for manganese and sensitize invaders to the manganese-withholding defense of the host, known as nutritional immunity. How pathogens manage these conflicting pressures is currently unknown. The current investigations revealed that a second metal-independent variant of phosphoglycerate mutase possessed by both *S*. *aureus* and *Salmonella* enables them to grow and consume glycolytic substrates in the presence of the manganese-binding immune effector calprotectin. Infection experiments revealed that the manganese-independent isozyme critically contributes to the ability of *S*. *aureus* to overcome manganese starvation during infection. Together, these results suggest that using metal-independent isozymes to enable the consumption of sugars within the host or other metal-limited environments is a common strategy employed by diverse bacteria.

## Introduction

The preferred carbon source for many pathogens is glucose and disruption of glycolysis reduces the ability of many invaders to cause infection [1–10]. The primacy of glucose as an energy source is emphasized by catabolite repression, which prevents bacteria from utilizing other carbon sources when glucose is present [1–3, 11–13]. The advantage that sugar consumption provides is highlighted by the increased sensitivity of individuals with diabetes, especially those with hyperglycemia, to infections by *Staphylococcus aureus*, *Streptococcus pneumoniae*, *Mycobacterium tuberculosis*, *Escherichia coli*, *Klebsiella pneumoniae* and *Candida albicans* [14–17]. At the same time, host defenses reduce the ability of pathogens to consume glycolytic substrates by limiting metal availability or via other mechanisms [18, 19]. The spread of antibiotic-resistant isolates has led the Centers for Disease Control and Prevention (CDC) and the World Health Organization (WHO) to call for the development of novel therapeutics to treat *S*. *aureus* and other pathogens [20, 21]. Understanding how pathogens generate energy and preserve the activity of critical metabolic pathways despite the concerted efforts of the immune system has the potential to identify new opportunities for therapeutic intervention.

Transition metals such as iron (Fe), manganese (Mn) and zinc (Zn) are essential for life, as they play an important role in facilitating the structure and function of proteins [22]. The host takes advantage of this essentiality by restricting the availability of Fe, Mn and Zn during infection, a defense known as nutritional immunity [23–28]. The prototypic example of Mn and Zn restriction is the staphylococcal abscess, which is virtually devoid of these metals [25, 29]. A key mediator of the host Mn-withholding response is the immune effector calprotectin (CP). A heterodimer of S100A8 and S100A9, CP possesses two transition metal binding sites that chelate Mn and Zn with nanomolar and femtomolar affinities, respectively [25, 27, 30–35]. Although CP binds other metals, including Fe (II) and nickel (Ni) [36, 37], the primary metals withheld from *S*. *aureus* are Mn and Zn. CP comprises ~50% of the total protein in the neutrophil cytoplasm and CP concentrations can exceed 1 mg/ml at sites of infection [38, 39]. Mice lacking CP have defects in Mn sequestration and are more sensitive to a range of bacterial and fungal pathogens, including *S*. *aureus*, *Acinetobacter baumannii*, *K*. *pneumoniae* and *Aspergillus fumigatus* [25–27, 40, 41].

During infection, nutritional immunity inactivates Mn-dependent bacterial processes such as the Mn-dependent superoxide dismutase possessed by *S*. *aureus*. This in turn renders *S*. *aureus* more sensitive to the oxidative burst of immune cells. While glycolysis is canonically believed to be a magnesium-dependent process [42], many bacteria possess Mn-dependent variants of glycolytic enzymes, including phosphoglycerate mutase, enolase and pyruvate kinase [43–50]. In fact, in a number of pathogens, including *S*. *aureus* and *S*. *pneumoniae*, the consumption of sugars is dependent on Mn availability [18, 51]. At the same time, glycolysis is critically important for the ability of *S*. *aureus* to cause infection and mutations that reduce the activity of this pathway frequently result in virulence defects and sensitize the bacterium to other host defenses such as NO· produced by immune cells [4–8]. While the best characterized mechanism utilized by bacteria to resist nutritional immunity is the use of high-affinity metal transporters, *S*. *aureus* and other pathogens also possess transporter-independent adaptations that critically contribute to the ability of the bacteria to overcome nutritional immunity [18, 29, 52–55]. However, mechanisms that would enable them to maximize the consumption of sugars via glycolysis when metal-starved have not been described.

Despite increasing the cellular demand for Mn and sensitizing *S*. *aureus* to Mn starvation, we recently observed that *S*. *aureus* prefers to consume glucose even when Mn-starved by CP [18, 55]. This paradoxical use of glycolysis despite the associated increase in cellular demand for Mn likely occurs in part as the fermentation of sugars enables the bacterium to generate energy when exposed to NO· produced by activated immune cells [8]. At the same time, it highlights a challenge faced by *S*. *aureus* and other pathogens, where adapting to one host defense sensitizes the bacterium to another aspect of the immune response [18, 55]. Many bacteria, including *S*. *aureus*, possess multiple copies of glycolytic enzymes. While the presence of multiple isozymes is frequently associated with glycolytic and gluconeogenic flux, we wondered if another reason for maintaining multiple isozymes might exist. Specifically, we hypothesized that multiple copies of glycolytic enzymes may help *S*. *aureus* and other pathogens mitigate the stress of consuming sugars when metal-starved. The current investigations revealed that expression of a second metal-independent variant of phosphoglycerate mutase enables *S*. *aureus* to maintain the ability to consume glucose when Mn-starved and critically contributes to resisting nutritional immunity during infection. Similar results were also observed with *Salmonella enterica*, suggesting that expression of metal-independent isozymes is a common strategy employed by bacteria to survive in metal-limited environments.

## Results

### Expression of metal-independent glycolytic enzymes increases in response to host-imposed metal starvation

**Fig 1:**
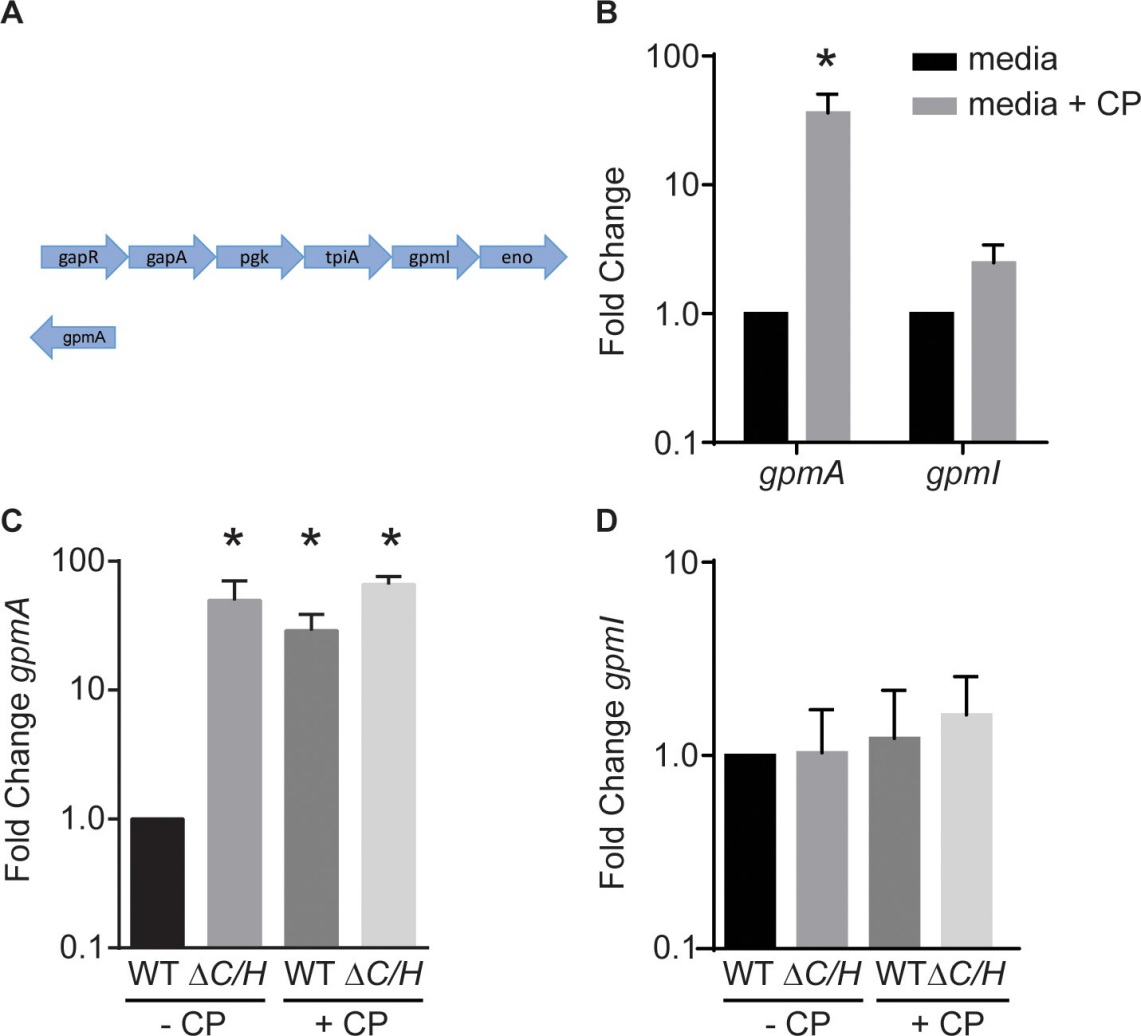
*S*. *aureus* induces a metal-independent phosphoglycerate mutase in response to calprotectin. (A) The chromosomal location of *gpmI* and *gpmA* is depicted. *gpmI* is part of the glycolytic operon, while *gpmA* is not part of an operon and is in a separate location. Not to scale. (B) Wild type *S*. *aureus* was grown in rich medium in the presence and absence of 240 μg/ml of CP and transcript levels of *gpmA* and *gpmI* were assessed by qRT-PCR. Expression was compared to wild type bacteria grown in the absence of CP. * = p≤0.05 relative to media alone by two-way ANOVA on log-transformed values with Tukey’s multiple comparisons test. (C-D) Wild type *S*. *aureus* and Δ*mntC*Δ*mntH* were grown in rich medium in the presence and absence of 240 μg/ml of CP and transcript levels of *gpmA* (C) and *gpmI* (D) were assessed by qRT-PCR. Expression was compared to wild type bacteria grown in the absence of CP. * = p≤0.05 relative to media alone by one-way ANOVA on log-transformed values with Tukey’s multiple comparisons test. Error bars indicate SEM. n≥3.

As an initial step to identify how *S*. *aureus* promotes retention of glycolysis during infection, the repertoire of glycolytic enzymes possessed by *S*. *aureus* was assessed. This analysis revealed that *S*. *aureus* possess two copies of glyceraldehyde-3-phosphate dehydrogenase, aldolase and phosphoglycerate mutase. Notably, one of the phosphoglycerate mutase isozymes, GpmI, is predicted to be Mn-dependent, while the other, GpmA, is Mn-independent, utilizing 2,3-bisphosphoglycerate as a catalytic cofactor [56, 57]. GpmI is encoded by the glycolytic operon that contains *gapR*, *gapA*, *pgk*, *tpiA* and *eno*, whereas *gpmA* is not part of an operon and is expressed independently of other glycolytic enzymes (Fig 1A). The fact that *gpmI* is in a locus with many other glycolytic enzymes, while *gpmA* is in a separate location, suggests that GpmI is the primary phosphoglycerate mutase and that GpmA is the secondary enzyme. One possibility is that GpmI and GpmA have directional preferences, with GpmI being essential for glycolysis and GpmA being essential for gluconeogenesis, as has been suggested for the two isozymes of glyceraldehyde-3-phosphate dehydrogenase encoded by *S*. *aureus* [58]. However, it is also possible that the metal-independent variant promotes retention of glycolytic flux when metal-starved by the host. As an initial step in evaluating this latter idea, expression of *gpmA* and *gpmI* in wild type bacteria exposed to CP was assessed. While expression of *gpmI* did not change, *gpmA* levels increased ~40-fold in response to CP (Fig 1B). Expression of *gpmA* was also induced in a *S*. *aureus* mutant lacking the Mn transporters MntABC and MntH (*ΔmntC*Δ*mntH*) to a level comparable to that caused by CP (Fig 1C). In contrast, expression of *gpmI* did not change in the *ΔmntC*Δ*mntH* mutant (Fig 1D). Cumulatively, these observations suggest that GpmI is the primary phosphoglycerate mutase used by *S*. *aureus* and that GpmA may be important when the bacteria experience Mn limitation.

### Expression of a metal-independent phosphoglycerate mutase promotes staphylococcal resistance to manganese starvation

**Fig 2:**
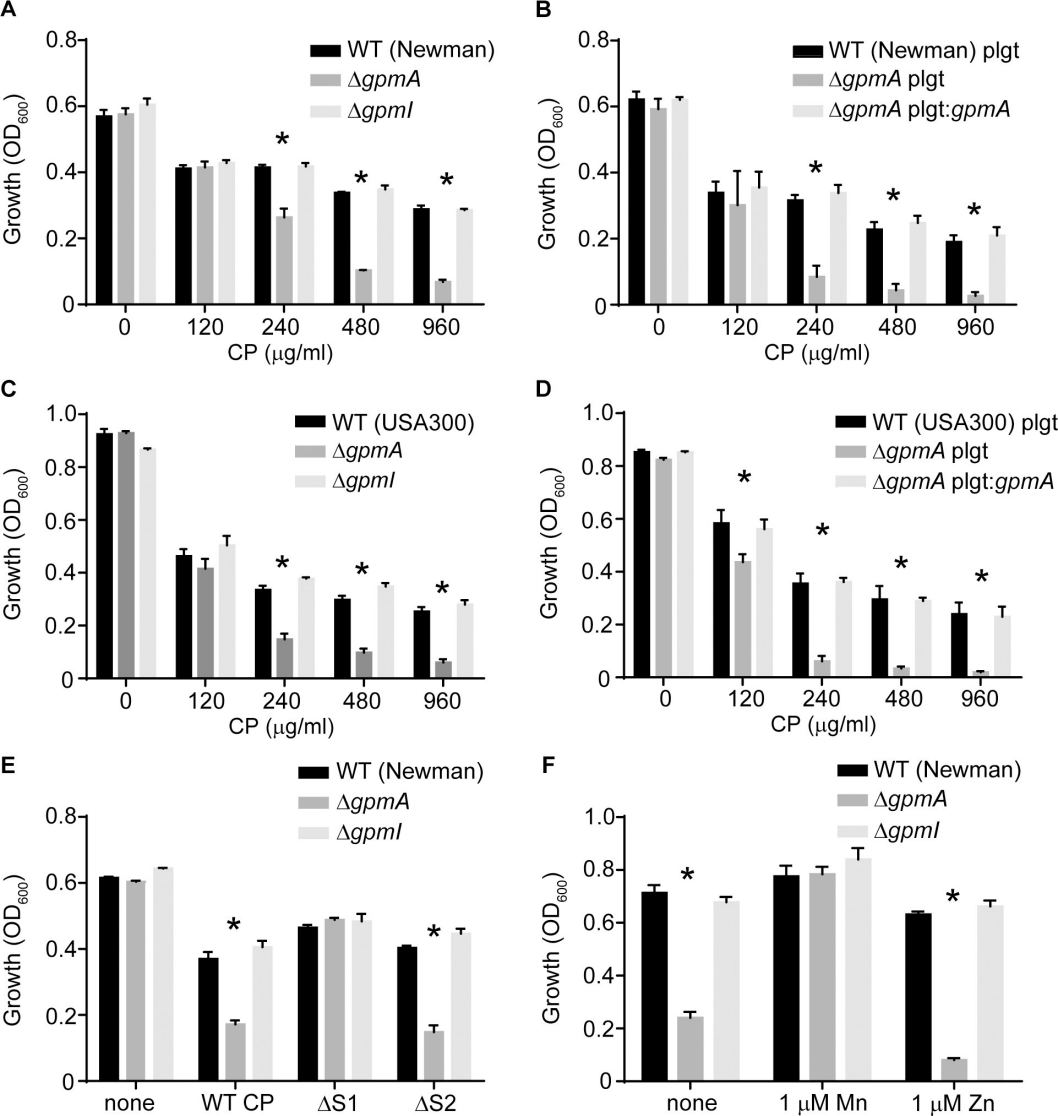
Expression of a metal-independent phosphoglycerate mutase promotes resistance to manganese starvation. (A) Wild type *S*. *aureus* Newman, Δ*gpmA* and Δ*gpmI* and (B) wild type *S*. *aureus* Newman and Δ*gpmA* containing either pOS1 plgt (plgt) or pOS1 plgt:*gpmA* (plgt:*gpmA*) were grown in rich medium in the presence of increasing concentrations of CP (t = 7). (C) Wild type USA300 JE2, Δ*gpmA* and Δ*gpmI* and (D) wild type USA300 JE2 and Δ*gpmA* containing either pOS1 plgt (plgt) or pOS1 plgt:*gpmA* (plgt:*gpmA*) were grown in rich medium in the presence of increasing concentrations of CP (t = 7). (E) Growth of wild type *S*. *aureus* Newman, Δ*gpmA* and Δ*gpmI* in the presence of 240 μg/ml of wild type CP as well as the ΔS1 and ΔS2 site mutants (t = 7). (F) Growth of wild type *S*. *aureus* Newman, Δ*gpmA* and Δ*gpmI* derivatives in NRPMI in the presence and absence of 1 μM MnCl_2_ or 1 μM ZnSO_4_ (t = 8). * = p≤0.05 relative to wild type and Δ*gpmI* by two-way ANOVA with Tukey’s multiple comparisons test. n≥3. Error bars indicate SEM. Also see S1 Fig.

To elucidate the respective contributions of the two staphylococcal phosphoglycerate mutases to resisting metal starvation, wild type as well as Δ*gpmA* and Δ*gpmI* mutants were evaluated for their ability to grow in the presence of CP. Loss of the metal-dependent isozyme, GpmI, in *S*. *aureus* Newman did not alter the sensitivity of *S*. *aureus* to CP. Conversely, loss of the metal-independent isozyme profoundly sensitized *S*. *aureus* to CP (Fig 2A, S1A Fig). Expression of GpmA from a plasmid reversed the increased sensitivity of Δ*gpmA* to CP (Fig 2B, S1B Fig). Increased sensitivity to CP was also observed upon loss of GpmA in the community-acquired MRSA strain USA300 JE2 (Fig 2C, S1C Fig), which was also reversed by expression of GpmA from a plasmid (Fig 2D, S1D Fig). Together, these results demonstrate that loss of GpmA makes *S*. *aureus* more sensitive to CP-imposed metal starvation.

To determine if Mn or Zn restriction was responsible for the increased sensitivity of Δ*gpmA* to CP, we leveraged CP binding site mutants with altered metal-binding properties [27, 32]. Similar to WT CP, when Δ*gpmA* was grown in the presence of the ΔS2 mutant, which can bind either Mn or Zn, it was more sensitive to CP treatment than wild type bacteria or Δ*gpmI* (Fig 2E). However, in the presence of the ΔS1 mutant, which cannot bind Mn, the increased sensitivity of Δ*gpmA* was abrogated. These observations suggest that loss of GpmA impairs the ability of the bacteria to cope with CP-induced Mn limitation. To further test this idea, wild type *S*. *aureus*, Δ*gpmA* and Δ*gpmI* were grown in medium depleted of Mn and Zn (NRPMI). Similar to what was observed in the presence of CP, the Δ*gpmA* mutant had a severe growth defect compared to wild type bacteria and the Δ*gpmI* mutant (Fig 2F, S1E Fig). The growth defect of the Δ*gpmA* mutant in this medium was reversed by the addition of Mn but not Zn. Collectively, these results demonstrate that GpmA is crucial for growth when *S*. *aureus* is Mn-starved.

### Expression of a metal-independent isozyme facilitates retention of glycolysis when Mn-starved

**Fig 3:**
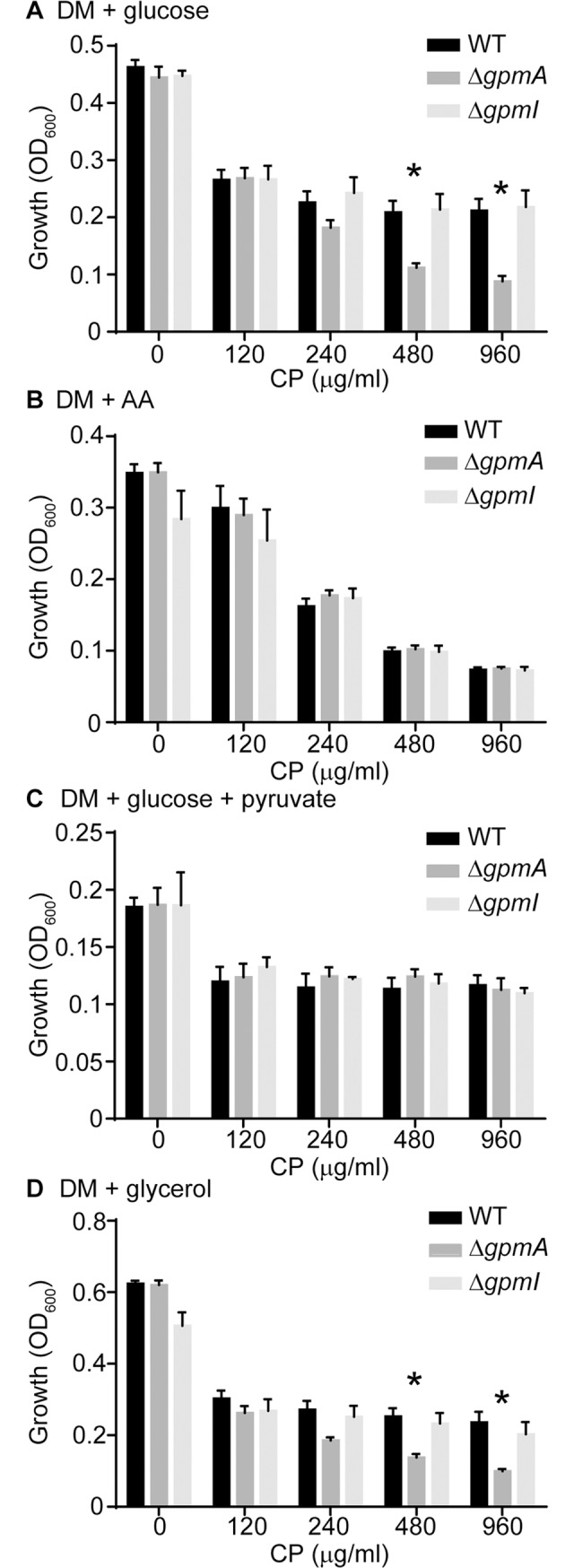
Expression of a metal-independent isozyme facilitates retention of glycolysis when manganese-starved. (A-D) *S*. *aureus* wild type, Δ*gpmA* and Δ*gpmI* were grown in defined medium supplemented with (A) glucose (DM + glucose), (B) Casamino acids (DM + AA), (C) glucose and sodium pyruvate (DM + glucose + pyruvate) or (D) glycerol (DM + glycerol) as a carbon source in the presence of increasing concentrations of CP (t = 7). * = p≤0.05 relative to wild type and Δ*gpmI* by two-way ANOVA with Tukey’s multiple comparisons test. n≥3. Error bars indicate SEM. Also see S2 Fig.

The majority of enzymes in the glycolytic pathway, including phosphoglycerate mutase, can be used for flux in both directions [59, 60]. To determine if the GpmA-dependent growth defect is associated with decreased glycolytic or gluconeogenic activity, wild type *S*. *aureus*, Δ*gpmA* and Δ*gpmI* were grown in the presence of CP in a defined medium supplemented with either glucose or Casamino acids as the sole energy source. In the presence of glucose, the Δ*gpmA* mutant was more sensitive to CP than wild type bacteria or Δ*gpmI* (Fig 3A, S2A Fig). In contrast, there was no difference between any of the strains when Casamino acids were provided (Fig 3B, S2B Fig). Supplementation of the glucose-containing medium with sodium pyruvate, which bypasses the phosphoglycerate mutase step, also enabled the Δ*gpmA* mutant to grow as well as wild type *S*. *aureus* or the Δ*gpmI* mutant (Fig 3C, S2C Fig). Together, these observations indicate that loss of GpmA reduces the ability of *S*. *aureus* to consume glucose when Mn-starved. To evaluate if this apparent defect in glycolysis extended to other substrates dependent on glycolysis for consumption, growth of the Δ*gpmA* and Δ*gpmI* mutants in defined medium supplemented with glycerol was assessed. In the presence of glycerol, which enters glycolysis upstream of phosphoglycerate mutase, as the sole carbon source the Δ*gpmA* mutant was more sensitive to CP than wild type bacteria or Δ*gpmI* (Fig 3D, S2D Fig). Combined, these results reveal that GpmA plays an important role in retaining the ability to consume glycolytic substrates when Mn-starved.

### Loss of GpmA does not affect manganese transporter function

**Fig 4:**
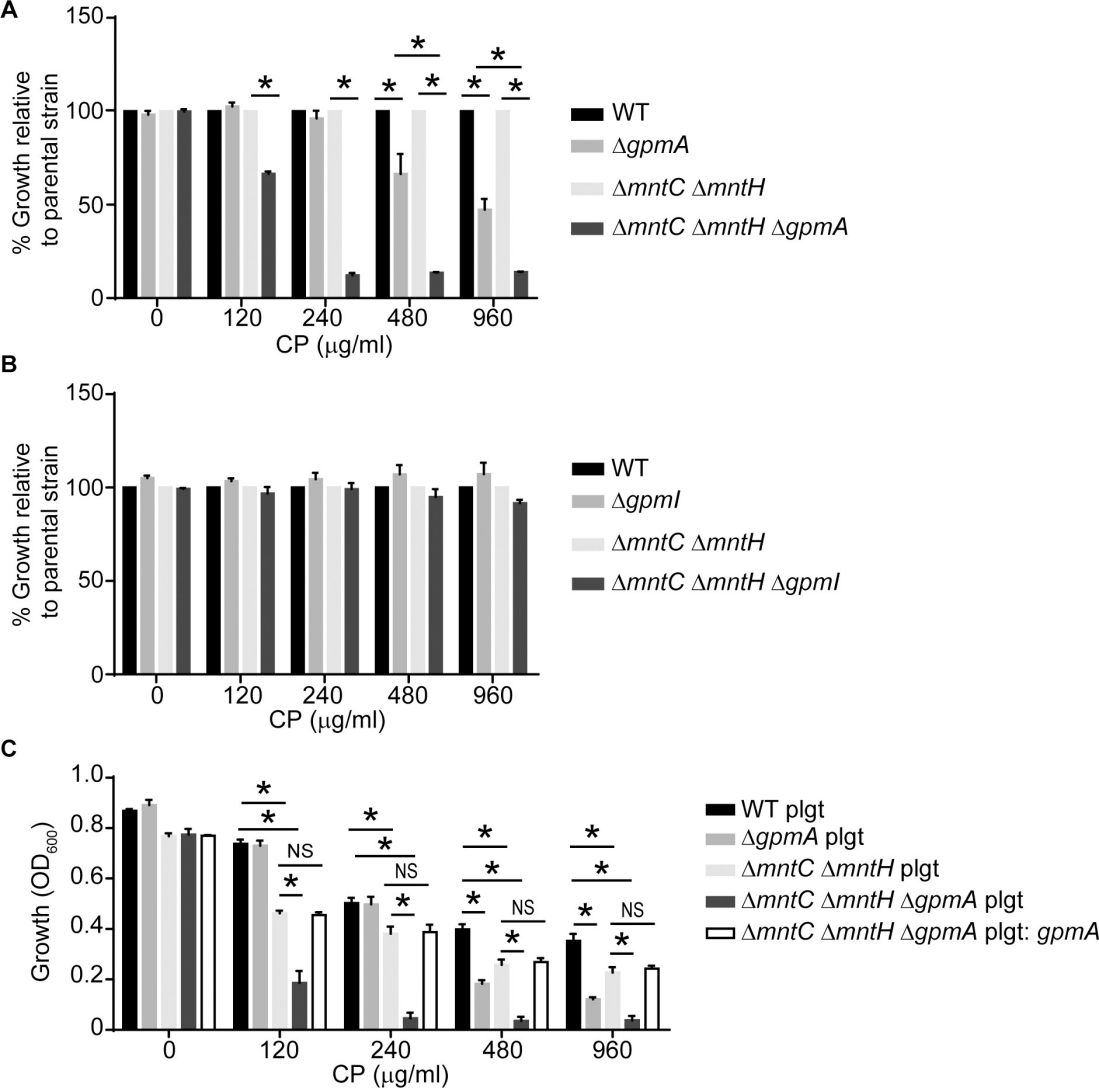
Loss of GpmA sensitizes *S*. *aureus* to manganese limitation in the absence of manganese transporters. The growth of (A) wild type, Δ*gpmA*, Δ*mntC* Δ*mntH* and Δ*mntC* Δ*mntH* Δ*gpmA* and (B) wild type, Δ*gpmI*, Δ*mntC* Δ*mntH* and Δ*mntC* Δ*mntH* Δ*gpmI* were assessed in rich medium supplemented with 1 μM MnCl_2_ and 1 μM ZnSO_4_ in the presence of increasing concentrations of CP (t = 8). For these assays, the bacteria were precultured overnight in TSB. * = p≤0.05 by two-way ANOVA with Tukey’s multiple comparisons test. n≥3. Error bars indicate SEM. Strains are normalized to growth of parental strains, i.e., Δ*gpmA* and Δ*gpmI* are normalized to wild type and Δ*mntC* Δ*mntH* Δ*gpmA* and Δ*mntC* Δ*mntH* Δ*gpmI* are normalized to Δ*mntC* Δ*mntH*. C) Wild type *S*. *aureus*, Δ*gpmA*, Δ*mntC* Δ*mntH*, Δ*mntC* Δ*mntH* Δ*gpmA* containing either pOS1 plgt (plgt) or pOS1 plgt: *gpmA* (plgt: *gpmA*) were grown in rich medium supplemented with 1 μM MnCl_2_ and 1 μM ZnSO_4_ in the presence of increasing concentrations of CP (t = 8). For these assays, the bacteria were precultured overnight in TSB. * = p≤0.05 by two-way ANOVA with Tukey’s multiple comparisons test. NS = not significant. n≥3. Error bars indicate SEM. Also see S3 Fig.

The expression of Mn transporters is critical for the ability of *S*. *aureus* to resist host-imposed Mn starvation [29]. To confirm that the enhanced sensitivity of the Δ*gpmA* mutant was not due to an unanticipated impact on Mn transporter activity, Δ*mntC*Δ*mntH*Δ*gpmA* and Δ*mntC*Δ*mntH*Δ*gpmI* strains were assessed for CP sensitivity. Loss of GpmA, but not GpmI, in the Δ*mntC*Δ*mntH* background further increased the sensitivity of the transporter double mutant, suggesting that GpmA and the Mn transporters function independently to promote resistance to Mn starvation (Fig 4A and 4B, S4A–S4C Fig). Expression of GpmA from a plasmid reverted the CP sensitivity of Δ*mntC*Δ*mntH*Δ*gpmA* back to that of the Δ*mntC*Δ*mntH* mutant (Fig 4C). Cumulatively, these results suggest that loss of GpmA does not sensitize the bacteria to Mn starvation by reducing Mn transport but rather by a Mn transporter-independent mechanism.

### GpmA is necessary for invasive *S*. *aureus* disease and resisting manganese starvation during infection

**Fig 5:**
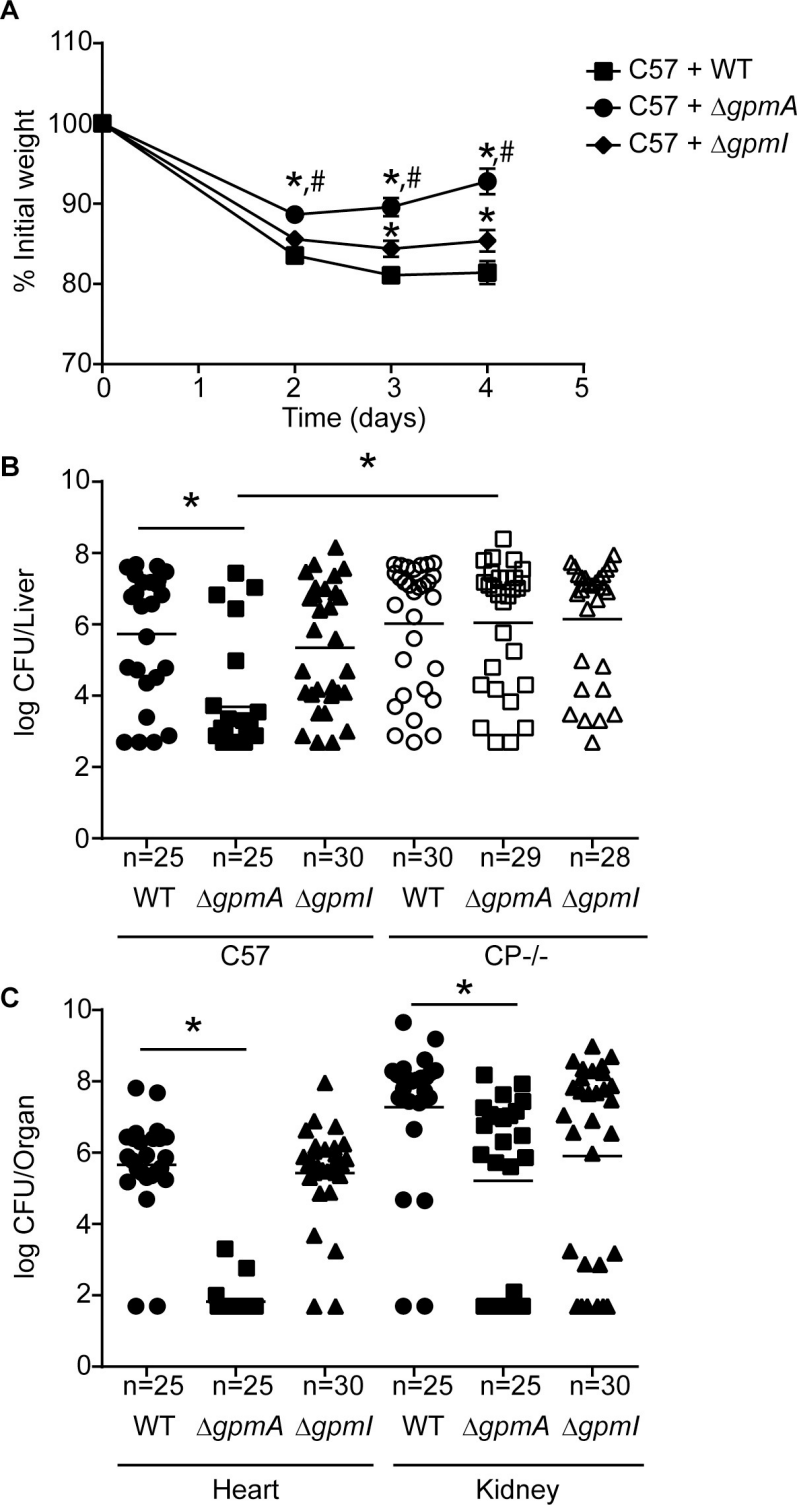
GpmA is necessary for invasive *S*. *aureus* disease and resisting manganese starvation. Wild type C57BL/6 (C57) and CP-deficient C57BL/6 S100A9-/- (CP-/-) mice were infected with either *S*. *aureus* wild type, Δ*gpmA* or Δ*gpmI* and (A) mean weight loss and (B-C) bacterial burdens in the (B) liver and (C) heart and kidneys were assessed after four days of infection. (A) *^,#^ = p≤0.05 as determined by two-way ANOVA with Tukey’s multiple comparisons test, with * compared to wild type bacteria and ^#^ compared to Δ*gpmI*. (B-C) * = p≤0.05 as determined by Mann-Whitney test for bacterial burdens. The lines indicate the mean. The data are the results from two independent experiments.

Culture-based experiments suggest that the Mn-independent activity of GpmA enhances the ability of *S*. *aureus* to maintain glycolytic flux when Mn-limited. To evaluate the contribution of the two phosphoglycerate mutases to *S*. *aureus* pathogenesis, wild type (C57BL/6) mice were retro-orbitally infected with wild type *S*. *aureus*, Δ*gpmA*, or Δ*gpmI* and the infection was allowed to proceed for 4 days. During the course of the infection mice infected with *ΔgpmA* lost significantly less weight than mice infected with wild type *S*. *aureus* or *ΔgpmI* (Fig 5A). Interestingly, mice infected with *ΔgpmI* lost slightly, but significantly, less weight than mice infected with wild type bacteria. Consistent with the weight loss, the *ΔgpmA* mutant had significantly decreased bacterial burdens in the liver, heart, and kidneys when compared to wild type bacteria (Fig 5B and 5C) indicating that GpmA plays an important role in establishing systemic disease. While mice infected with *ΔgpmI* did not show a statistically significant decrease in bacterial burdens in any of the organs, bacterial burdens in the kidneys were slightly lower than in mice infected with wild type bacteria (Fig 5C). To evaluate if the importance of GpmA during infection is driven by host restriction of Mn availability, CP-deficient (C57BL/6 S100A9-/-) mice, which do not remove Mn from liver abscesses [25, 29], were infected with wild type bacteria, *ΔgpmA* or *ΔgpmI*. Relative to wild type mice, the CP-deficient mice infected with *ΔgpmA* had increased bacterial burdens, indicating that the importance of GpmA during infection is driven by host-imposed Mn limitation. Moreover, in CP-deficient mice there was no difference in bacterial burdens between wild type bacteria, *ΔgpmA* or *ΔgpmI* (Fig 5B), suggesting either phosphoglycerate mutase isozyme is sufficient when Mn is available. Cumulatively, these results indicate that GpmA contributes to staphylococcal infection by promoting retention of glycolytic activity when Mn-starved by the host.

### A metal-independent phosphoglycerate mutase enables glycolysis when *Salmonella* is manganese-starved

**Fig 6:**
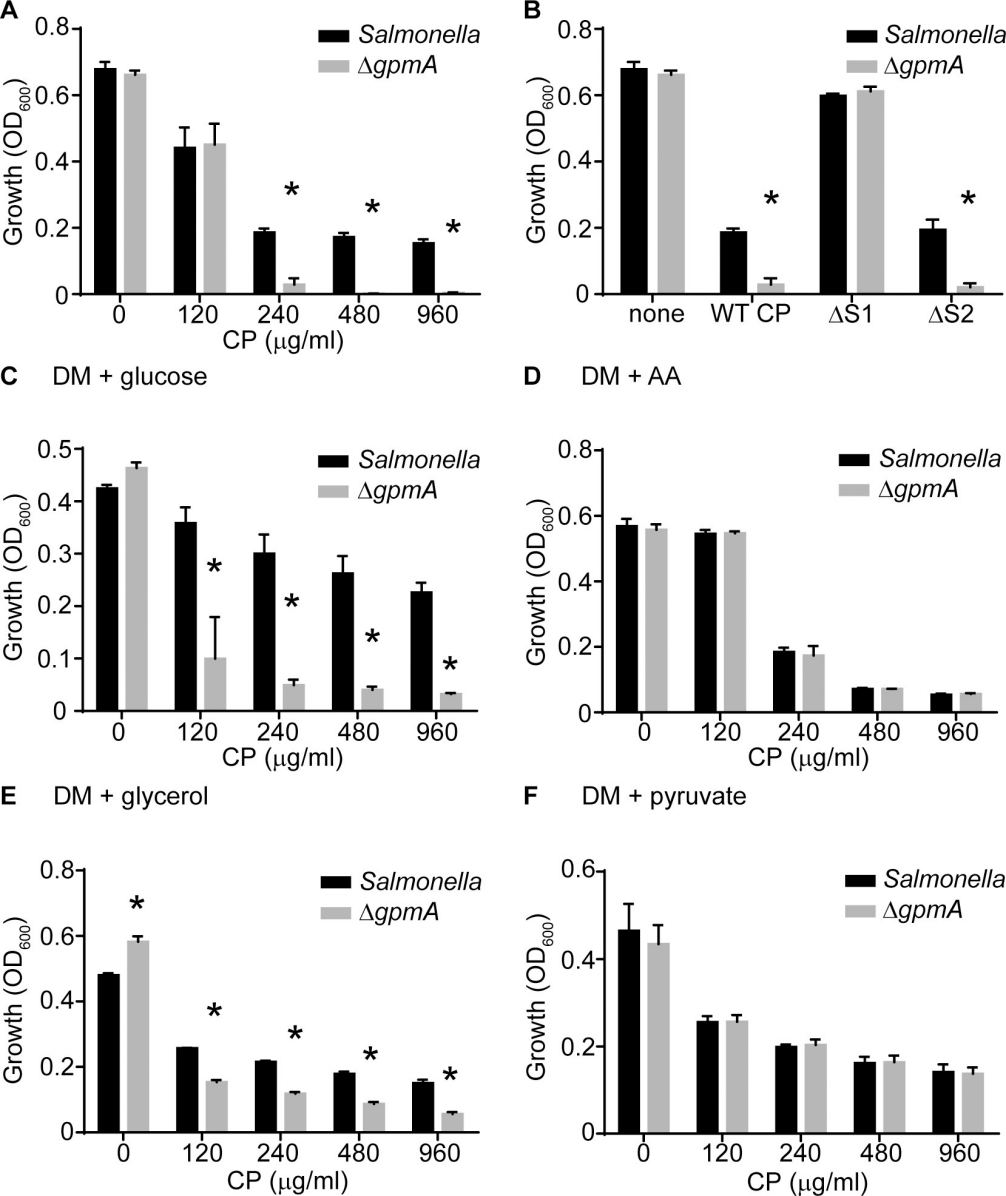
A metal-independent phosphoglycerate mutase enables glycolysis when *S*. *enterica* serovar Typhimurium is manganese-starved. (A & B) Wild type *Salmonella* and Δ*gpmA* were grown in rich medium in the presence of (A) increasing concentrations of CP or (B) 240 μg/ml of WT CP or the ΔS1 and ΔS2 CP mutants (t = 8). (C-F) Wild type *Salmonella* and Δ*gpmA* were grown in defined medium (DM) supplemented with (C) glucose (DM + glucose), (D) Casamino acids (DM + AA), (E) glycerol (DM + glycerol) or (F) sodium pyruvate (DM + pyruvate) as a carbon source in the presence of increasing concentrations of CP (t = 8). * = p≤0.05 relative to wild type by two-way ANOVA with Sidak’s multiple comparisons test. n≥3. Error bars indicate SEM. Also see S4 Fig.

The expression of metal-dependent and -independent variants of phosphoglycerate mutase is not unique to *S*. *aureus*, and many pathogenic bacteria including *E*. *coli*, *Shigella flexneri*, *S*. *enterica*, *Pseudomonas aeruginosa*, *Listeria monocytogenes* and others possess both forms [57]. However, the molecular rationale for retaining two phosphoglycerate mutase isozymes in these organisms remains unknown [57]. In light of our observation with *S*. *aureus*, we wondered if metal-independent variants of phosphoglycerate mutase broadly promote retention of glycolytic potential when experiencing Mn starvation. To test this idea, the ability of wild type *S*. *enterica* serovar Typhimurium and a *ΔgpmA* mutant to grow in rich medium in the presence of CP was assessed. Similar to *S*. *aureus*, the *Salmonella ΔgpmA* mutant was more sensitive to CP treatment than wild type bacteria (Fig 6A, S4A Fig). Use of the CP variants with altered metal-binding properties revealed that loss of GpmA impaired the ability of *Salmonella* to cope with CP-induced Mn limitation (Fig 6B). Cumulatively, these observations establish that expression of a Mn-independent variant of phosphoglycerate mutase promotes the growth of *Salmonella* when metal-starved.

To test if the importance of GpmA to *Salmonella* growth when metal-starved is attributable to its ability to promote consumption of glycolytic substrates, bacteria were grown in the presence of CP and provided with either glucose or Casamino acids as a carbon source (Fig 6C and 6D, S4B and S4C Fig). As in rich medium, loss of GpmA increased the sensitivity of *Salmonella* to CP when glucose was provided as the sole carbon source. However, the enhanced sensitivity of the *Salmonella ΔgpmA* mutant was ablated in the presence of Casamino acids, which do not require the glycolytic pathway for consumption. Additionally, providing sodium pyruvate as a carbon source reversed the increased sensitivity to CP, whereas glycerol did not (Fig 6E and 6F, S4D and S4E Fig). Combined, these results suggest that the expression of metal-independent versions of phosphoglycerate mutase are a common mechanism employed by pathogenic bacteria to resist Mn limitation.

## Discussion

The consumption of sugars via glycolysis and metals are important for pathogens during infection [4, 5, 7–10, 24, 61–66]. Sugars are the preferred carbon source for *S*. *aureus* and other pathogens and increased availability of glucose renders individuals more sensitive to infection [14–17]. At the same time, the host restricts the availability of metals, inactivating Mn-dependent bacterial processes, including glycolysis [18, 55]. While expression of high-affinity metal transporters enhances the ability of *S*. *aureus* and other pathogens to maintain glycolytic flux and are critical for infection, they are insufficient to ensure that the pathogen obtains sufficient Mn to activate Mn-dependent superoxide dismutases [27, 53]. The current work provides yet another example of a critical Mn-dependent enzyme, GpmI, which is inactivated by nutritional immunity. At the same time, host defenses force *S*. *aureus* to ferment sugars via the glycolytic pathway, increasing the cellular demand for this metal [5, 7, 8]. The current work identified a critical role for *a priori* redundant metal-independent variants of phosphoglycerate mutases in both *S*. *aureus* and *Salmonella* in enabling glucose consumption and resisting nutritional immunity. The Mn-independent variant of phosphoglycerate mutase in *S*. *aureus*, which is encoded outside of the canonical glycolytic operon, is the primary isozyme used by the bacteria when Mn-starved and is critical for infection due to the host metal-withholding response. The conserved function of the metal-independent variant of phosphoglycerate mutase in both *S*. *aureus* and *Salmonella* suggests that this approach is likely a common strategy for preserving the ability to consume glucose while minimizing the cellular demand for Mn.

*S*. *aureus* and *Salmonella* are not the only pathogens that experience metal limitation during infection nor are they the only pathogens to require glucose consumption for disease [1, 5–10, 28, 65, 66]. It therefore seems likely that other successful pathogens have adaptations that allow them to consume glucose when metal-limited. Notably, in addition to *S*. *aureus* and *Salmonella*, many other bacterial pathogens, including *S*. *flexneri*, *P*. *aeruginosa*, and *L*. *monocytogenes* possess metal-dependent and -independent variants of phosphoglycerate mutase [57]. As CP is one of the most abundant proteins at sites of infection [38, 39], it seems likely that all of these pathogens experience Mn limitation during infection. Combined with the current observations, this suggest that using metal-independent variants of phosphoglycerate mutase to preserve glycolytic function in the host when Mn-starved may be a conserved strategy.

Intriguingly, a number of bacterial pathogens, including *S*. *pneumoniae*, *Enterococcus faecalis*, *Haemophilus influenzae*, *Neisseria meningiditis*, and *M*. *tuberculosis* possess only a metal-independent version of phosphoglycerate mutase [56, 57]. Similar to other pathogens, all of these organisms would be expected to encounter Mn-limited environments within the host. This raises the possibility that possessing only the metal-independent variant of phosphoglycerate mutase represents further adaptation to life in a Mn-poor environment. At the same time, the majority of bacteria, archaea, protozoa and fungi, including pathogens, possesses both metal-dependent and -independent versions of phosphoglycerate mutase. However, others, such as *Helicobacter pylori*, possesses only the metal-dependent variant but also encounter CP during infection [67]. The biochemical differences between the metal-dependent and independent-variants of phosphoglycerate mutase extend beyond their cofactor [47, 56, 68–70]. Notably, in *S*. *aureus* the metal-dependent variant is in the primary glycolytic operon. Together, these observations suggest that the metal-dependent variants of phosphoglycerate mutase provide some biological advantage.

Phosphoglycerate mutase is not the only enzyme in the glycolytic pathway that contains both metal-dependent and -independent versions. Both metal-dependent and -independent variants of fructose bisphosphate aldolase exist, and many pathogenic bacteria, including *S*. *aureus*, *Salmonella*, *Borrelia burgdorferi*, *S*. *pneumoniae*, and *K*. *pneumoniae* contain both enzymes. The metal-dependent variant is classically thought to utilize Zn as a cofactor [71–73]. Similar to GpmA, which is upregulated in response to CP treatment, the metal-independent staphylococcal aldolase is also upregulated in the presence of CP [74]. As aldolase is the only staphylococcal enzyme in glycolysis predicted to use Zn, this could explain why CP-imposed Mn limitation but not Zn limitation inactivates glycolysis in *S*. *aureus* [18, 55]. More broadly, it suggests that the metal-independent fructose bisphosphate aldolase may contribute to retaining glycolytic function when metal-starved by the host. In both *Salmonella* and *B*. *burgdorferi*, the Zn-independent aldolase has been suggested to provide a mechanism for the bacteria to consume glucose when experiencing nitrosative stress [19]. In the case of *B*. *burgdorferi*, nitrosative stress was suggested to damage the Zn-dependent isozyme [19]. At the same time, in *S*. *aureus* nitrosative stress has been observed to lead to a transcriptional response similar to that seen when Zn-starved [74]. Regardless of the stress that the metal-independent aldolase responds to, these observations suggest that metal-independent isozymes are likely to be generally important to the ability of bacteria to maintain glycolytic flux during infection.

The presence of two isozymes with differing metal utilization is not limited to glycolysis or *S*. *aureus* and *Salmonella*. In *Bacillus subtilis*, Fe limitation leads to induction of the Fe-sparing response, which includes increased expression of flavodoxin that can replace the iron-containing electron transfer protein ferredoxin [75]. Similarly, *E*. *coli* contains two ribonucleotide reductases, a primary Fe-dependent enzyme, and a Mn-dependent enzyme that is crucial for survival when bacteria experience superoxide stress or Fe limitation [76, 77]. Similarly, in response to Zn limitation, *B*. *subtilis* will induce expression of an alternative Zn-independent folate biosynthesis enzyme, GTP cyclohydrolase-IB (GCYH-IB). Induction of GCYH-IB prevents Zn starvation from inducing a folate auxotrophy [78]. *B*. *subtilis* also possesses L31* and L33*, Zn-independent ribosomal proteins, which promote survival when Zn-starved [79, 80]. Combined with the current observations, this suggests that replacing metal-dependent enzymes with metal-independent variants is a common strategy for surviving Fe, Zn and Mn limitation. Notably, the switch between utilizing the metal-dependent and -independent isozyme is frequently driven by a metal-sensing regulator [75, 76, 78–80]. In *S*. *aureus*, loss of the Fe-sensing regulator represses the expression of *gpmA*, but it is not predicted to be regulated by the Mn-sensing regulator MntR [81]. While the mechanisms that control expression of GpmA are unknown, this suggests that metal-independent regulatory circuits also play an important role in coordinating a response to metal starvation. This idea is supported by the observation that ArlRS, which contributes to staphylococcal growth in Mn-poor environments, appears not to sense Mn directly but rather the impact that Mn starvation has on glycolytic flux (*Parraga et*. *al*. In Press).

The current investigations add metal-dependent and -independent phosphoglycerate mutases to the growing list of enzymes that carry out apparently redundant biochemical reactions, but whose distinct reaction mechanisms enable microbes to cause infection or survive in other stressful environments [5, 6, 19, 75, 76, 78–80]. Many of these false redundancies have been identified by studying how microbes respond to metal limitation [75–80]. However, the importance of other pseudo-redundant enzymes has been revealed by investigating how pathogens cope with other stresses experienced during infection, such as the contribution of a second staphylococcal lactic acid dehydrogenase to growth in the presence of nitric oxide and infection [5, 6, 19]. While diverse stresses have been examined, a common denominator in all of these studies is that they have pushed the microbes outside of their ideal environments. As the importance of physiology to pathogenesis continues to be revealed, the current and prior studies reveal the importance of considering the environment encountered within the host when evaluating the contribution of enzymes to metabolism and infection.

## Materials and methods

### Ethics statement

All experiments involving animals were reviewed and approved by the Institutional Animal Care and Use Committee of the University of Illinois Urbana-Champaign (IACUC license number 15059) and performed according to NIH guidelines, the Animal Welfare Act, and US Federal law.

### Bacterial strains

Bacteria were routinely grown on tryptic soy agar (TSA) plates. For routine overnight cultures, bacteria were grown in 5 ml of tryptic soy broth (TSB) or in Chelex-treated RPMI plus 1% Casamino acids (NRPMI) supplemented with 1 mM MgCl_2_, 100 μM CaCl_2_ and 1 μM FeCl_2_ [29] in 15 ml conical tubes at 37°C on a roller drum. As needed, 10 μg/ml of chloramaphenicol was added for plasmid maintenance. *S*. *aureus* strain Newman and its derivatives were used for all of the experiments, unless otherwise indicated. For experiments using USA300 JE2 and derivatives (USA300 JE2 *gpmA*:erm and USA300 JE2 *gpmI*:erm), strains were obtained from the Nebraska library [82]. *gpmA*:erm, *gpmI*:erm, Δ*mntC*Δ*mntH gpmA*:erm and Δ*mntC*Δ*mntH gpmI*:erm were generated by transducing the *gpmA*:erm and *gpmI*:erm alleles via Φ85 phage from USA300 JE2 *gpmA*:erm and USA300 JE2 *gpmI*:erm. As needed 500 μM MnCl_2_ was added to agar plates to facilitate recovery of mutants. All constructs were confirmed to be hemolytic by growth on TSA blood agar plates. To generate constructs for complementation studies, the *gpmA* coding sequence was amplified with the indicated primers (Table 1) and cloned into the pOS1 vector under the control of the P_lgt_ promoter [83].

**Table 1:** PCR primers used in this study.

Name	Sequence
GpmA 5’ Comp	AGTCCATATGCCAAAATTAATTTTATGTCGTC
GpmA 3' Comp	AGTCGGATCCTTATAAGTAGTATTTATC
16S rRNA-F	GCTGCAGCTAACGCATTAAGCACT
16S rRNA-R	TTAAACCACATGCTCCACCGCTTG
GpmA F	GCGTTTAAATGAACGCCACT
GpmA R	CACGTTGTTCTTCGGTTTCA
GpmI F	AGAGCAGCGCAATTATCGGA
GpmI R	TCGAAGACGATAGCCGCATC
K.O. gpmA F	ATGATTTATAGGAGTGAGAGTTGTAGGCTGGAGCTG
K.O. gpmA R	TATACCGCCATCCGGCAAAGGTGACATATGAATATCCTC

*S*. *enterica* serovar Typhimurium strain 14028 was used for all *Salmonella* experiments. The deletion of *gpmA* by inserting a chloramphenicol cassette was carried out using lambda red-mediated recombination as described previously using the indicated primers (Table 1) [84, 85]. The insertion of the cassette was checked by PCR analysis and the construct was moved into a clean background by P22 transduction.

### CP growth assays

CP growth assays were performed as described previously [27, 32]. Briefly, overnight cultures (grown 16–18 h at 37°C on a roller drum) were used directly and diluted 1:100 into 96-well round-bottom plates containing 100 μl of growth medium (38% TSB and 62% calprotectin buffer (20 mM Tris pH 7.5, 100 mM NaCl, 3 mM CaCl_2_, 10 mM β-mercaptoethanol)) in presence of varying concentrations of CP. Unless otherwise indicated, the bacteria were grown overnight in NRPMI supplemented with 1 mM MgCl_2_, 100 μM CaCl_2_ and 1 μM FeCl_2_ and directly inoculated 1:100 into the assay medium. For all assays, the bacteria were incubated with orbital shaking (180 RPM) at 37°C and growth was measured by assessing optical density (OD_600_) every 2 hours. Prior to measuring optical density, the 96-well plates were vortexed. For experiments utilizing defined medium, the bacteria were precultured overnight in TSB. The defined medium consisted of 1.3 g/L NaCl, 2.6 g/L NH_4_Cl, 5.2 g/L KH_2_PO_4_, 18.2 g/L Na_2_HPO_4_, 0.593 μg/L biotin, 0.593 mg/L nicotinic acid, 0.593 mg/L pyridoxine-HCl, 0.593 mg/L thiamine-HCl, 0.296 mg/L riboflavin, 1.778 g/L calcium pantothenate, 0.104 g/L phenylalanine, 0.078 g/l isoleucine, 0.13 g/l tyrosine, 0.053 g/L cysteine, 0.26 g/L glutamic acid 0.026 g/L lysine, 0.182 g/L methionine, 0.078 g/L histidine, 0.026 g/L tryptophan, 0.234 g/L leucine, 0.234 g/L aspartic acid, 0.182 g/L arginine, 0.078 g/L serine, 0.15 g/L alanine, 0.078 g/L threonine, 0.130 g/L glycine, 0.208 g/L valine and 0.026 g/L proline. The defined medium was supplemented with 6 mM MgSO_4_, 1 μM FeCl_2_, 1 μM MnCl_2_ and 1 μM ZnSO_4_. Casamino acids (6.5%), glucose (1.3%), glycerol (1.3%), glucose (1.3%) + sodium pyruvate (0.22%) or sodium pyruvate (1.3%) only were provided as carbon sources as indicated. Calprotectin was purified as previously described [27, 32]. For growth assays in NRPMI, bacteria were grown overnight in NRPMI supplemented with 1 mM MgCl_2_, 100 μM CaCl_2_ and 1 μM FeCl_2_ and directly inoculated 1:100 into the assay medium. The assay medium consisted of NRPMI and the bacteria were grown in the presence and absence of 1 μM MnCl_2_ or 1 μM ZnSO_4_. For CP growth assays with *Salmonella*, 5 mM β-mercaptoethanol was used.

### Expression analysis

To assess the expression of *gpmA* and *gpmI*, *S*. *aureus* was grown as for CP growth assays in complex medium in the presence and absence of 240 μg/ml of CP, with the exception that they were precultured overnight in TSB. Bacteria were harvested during log phase growth (OD_600_ = ~0.1), when the samples were collected, an equal volume of ice-cold 1:1 acetone-ethanol was then added to the cultures, and they were frozen at -80°C until RNA extraction. RNA was extracted and cDNA was generated, as previously described [86–88]. Gene expression was assessed by quantitative reverse transcription-PCR (qRT-PCR) using the indicated primers (Table 1, [29]) and 16S was used as a normalizing control.

### Animal infections

Mouse infections were performed as described previously [25, 27]. Briefly, 9-week old wild type or CP-deficient (S100A9-/-) mice were infected retro-orbitally with approximately 5 x 10^6^ CFU in 100 μl of sterile phosphate-buffered saline. Following injection, the infection was allowed to proceed for 96 h before the mice were sacrificed. Livers, hearts and kidneys were removed, the organs were homogenized, and bacterial burden was determined by plating serial dilutions.

## Supporting information

S1 Fig Expression of a metal-independent phosphoglycerate mutase promotes resistance to manganese starvation. Growth curves for the data presented in **Fig** 2. (A) Wild type *S*. *aureus* Newman, Δ*gpmA* and Δ*gpmI* and (B) wild type *S*. *aureus* Newman and Δ*gpmA* containing either pOS1 plgt (plgt) or pOS1 plgt:*gpmA* (plgt:*gpmA*) were grown in rich medium in the presence of increasing concentrations of CP. (C) Wild type USA300, Δ*gpmA* and Δ*gpmI* and (D) wild type USA300 and Δ*gpmA* containing either pOS1 plgt (plgt) or pOS1 plgt:*gpmA* (plgt:*gpmA*) were grown in rich medium in the presence of increasing concentrations of CP. (E) Growth of wild type *S*. *aureus* Newman, Δ*gpmA* and Δ*gpmI* derivatives, in NRPMI in the presence and absence of 1 μM MnCl_2_ or 1 μM ZnSO_4_.
(TIF)

S2 Fig Expression of a metal-independent isozyme facilitates retention of glycolysis when manganese-starved. Growth curves for the data presented in **Fig** 3. (A-D) *S*. *aureus* wild type, Δ*gpmA* and Δ*gpmI* were grown in defined medium supplemented with (A) glucose (DM + glucose), (B) Casamino acids (DM + AA), (C) glucose and sodium pyruvate (DM + glucose + pyruvate) or (D) glycerol (DM + glycerol) as a carbon source in the presence of increasing concentrations of CP.
(TIF)

S3 Fig Loss of GpmA sensitizes *S. aureus* to manganese limitation in the absence of manganese transporters. Growth curves for the data presented in **Fig** 4. (A & B) The growth of wild type, Δ*gpmA*, Δ*mntC* Δ*mntH* and Δ*mntC* Δ*mntH* Δ*gpmA*, Δ*gpmI*, Δ*mntC* Δ*mntH* and Δ*mntC* Δ*mntH* Δ*gpmI* were assessed in rich medium supplemented with 1 μM MnCl_2_ and 1 μM ZnSO_4_ in the presence of increasing concentrations of CP. Panel A shows the optical density of wild type mutant strains at t = 8 before normalization to either wild type or the Δ*mntC* Δ*mntH* background.
(TIF)

S4 Fig A metal-independent phosphoglycerate mutase enables glycolysis when *S. enterica* Typhimurium is manganese-starved. Growth curves for the data presented in **Fig** 6. (A) Wild type *Salmonella* and Δ*gpmA* were grown in rich medium in the presence of increasing concentrations of CP. (B-E) Wild type *Salmonella* and Δ*gpmA* were grown in defined medium (DM) supplemented with (B) glucose (DM + glucose), (C) Casamino acids (DM + AA), (D) glycerol (DM + glycerol) or (E) sodium pyruvate (DM + pyruvate) as a carbon source in the presence of increasing concentrations of CP.
(TIF)
