# Supplementary figures and images for: Metal-independent variants of phosphoglycerate mutase promote resistance to nutritional immunity and retention of glycolysis during infection
Source: PLoS Pathog. 2019 Jul 25;15(7):e1007971. doi: 10.1371/journal.ppat.1007971 (PMC6684088; doi:10.1371/journal.ppat.1007971)

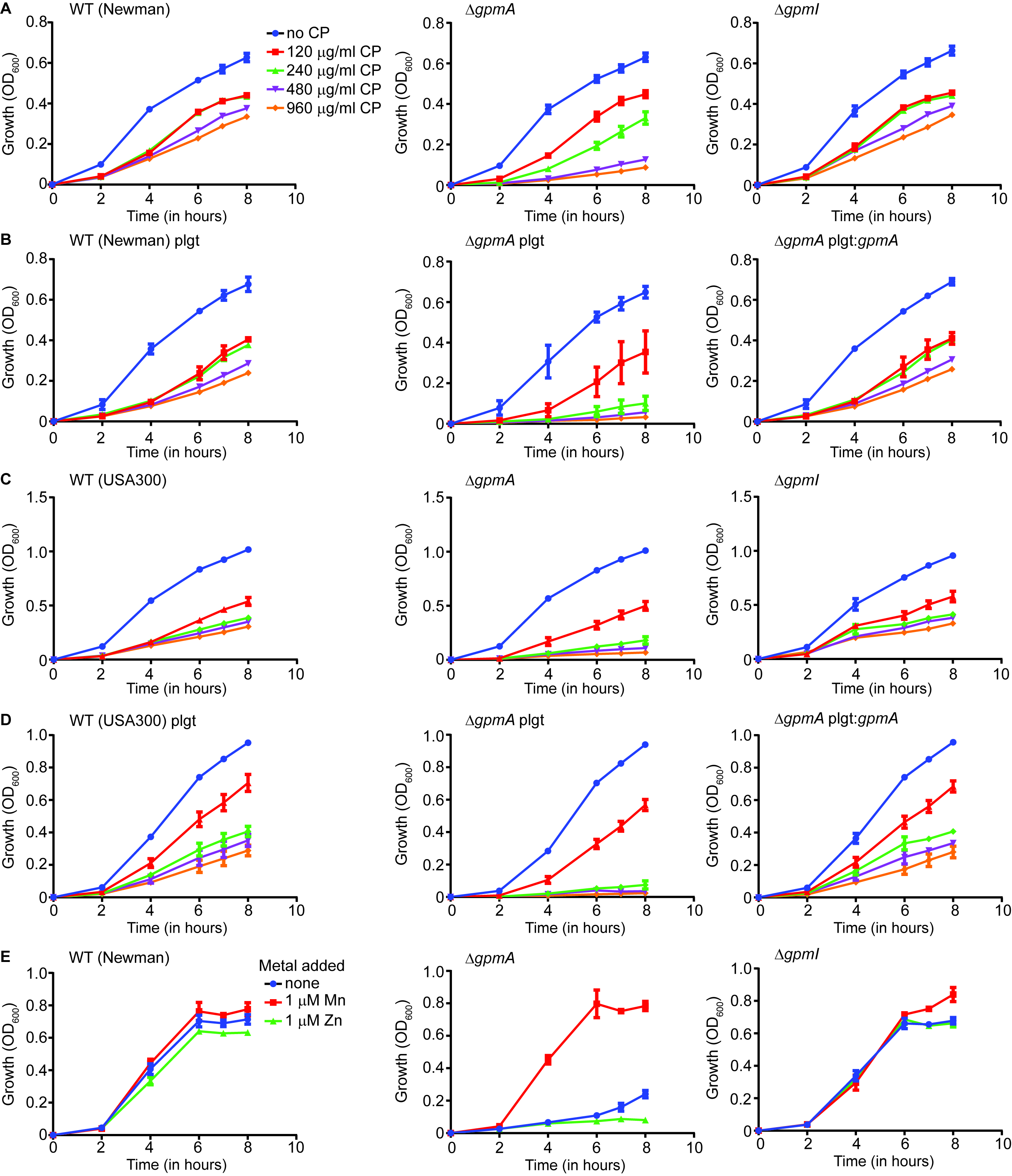

Supplement: S1 Fig — Growth curves for the data presented in Fig 2. (A) Wild type S. aureus Newman, ΔgpmA and ΔgpmI and (B) wild type S. aureus Newman and ΔgpmA containing either pOS1 plgt (plgt) or pOS1 plgt:gpmA (plgt:gpmA) were grown in rich medium in the presence of increasing concentrations of CP. (C) Wild type USA300, ΔgpmA and ΔgpmI and (D) wild type USA300 and ΔgpmA containing either pOS1 plgt (plgt) or pOS1 plgt:gpmA (plgt:gpmA) were grown in rich medium in the presence of increasing concentrations of CP. (E) Growth of wild type S. aureus Newman, ΔgpmA and ΔgpmI derivatives, in NRPMI in the presence and absence of 1 μM MnCl2 or 1 μM ZnSO4. (TIF) [file ppat.1007971.s001.tif]

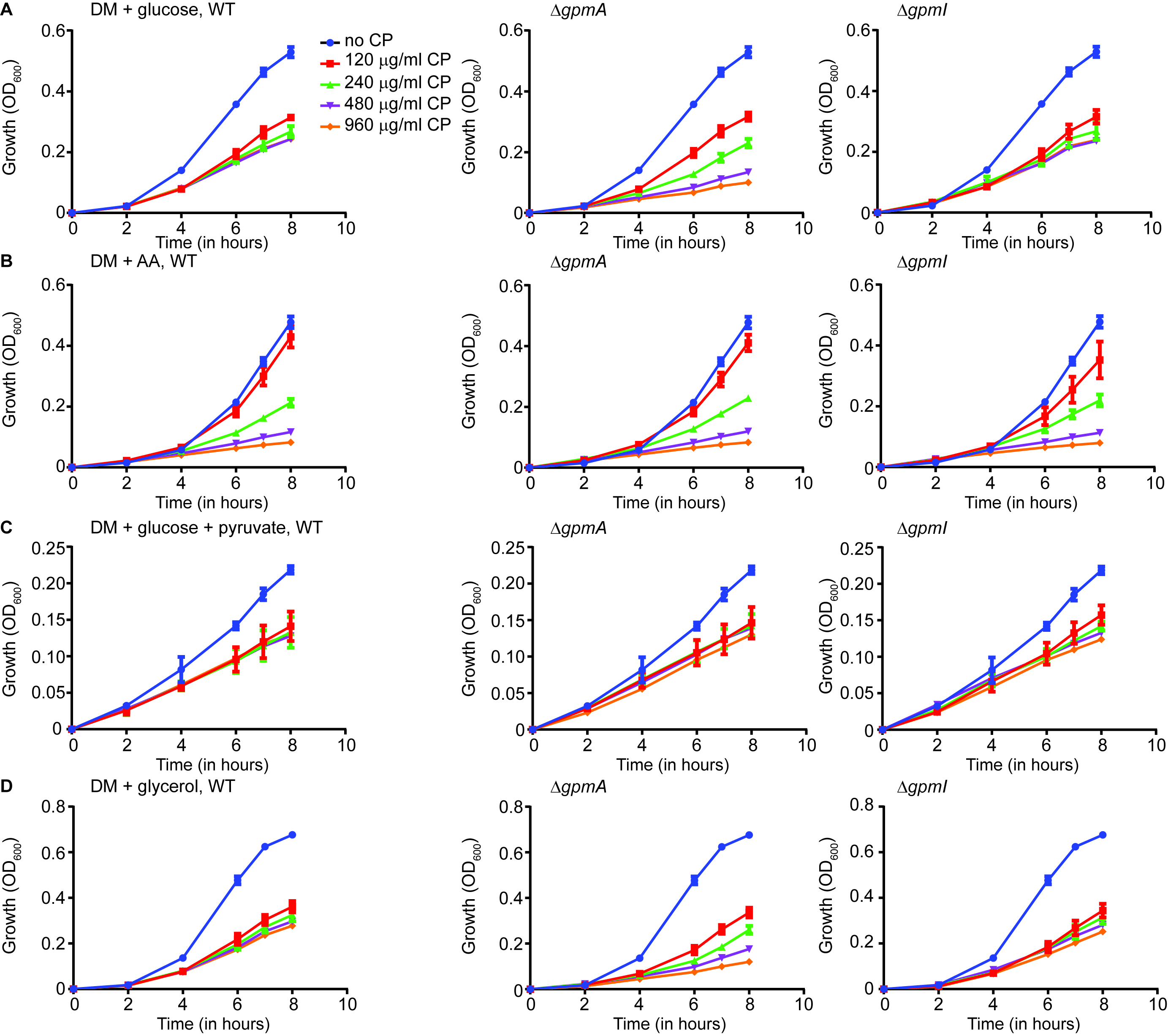

Supplement: S2 Fig — Growth curves for the data presented in Fig 3. (A-D) S. aureus wild type, ΔgpmA and ΔgpmI were grown in defined medium supplemented with (A) glucose (DM + glucose), (B) Casamino acids (DM + AA), (C) glucose and sodium pyruvate (DM + glucose + pyruvate) or (D) glycerol (DM + glycerol) as a carbon source in the presence of increasing concentrations of CP. (TIF) [file ppat.1007971.s002.tif]

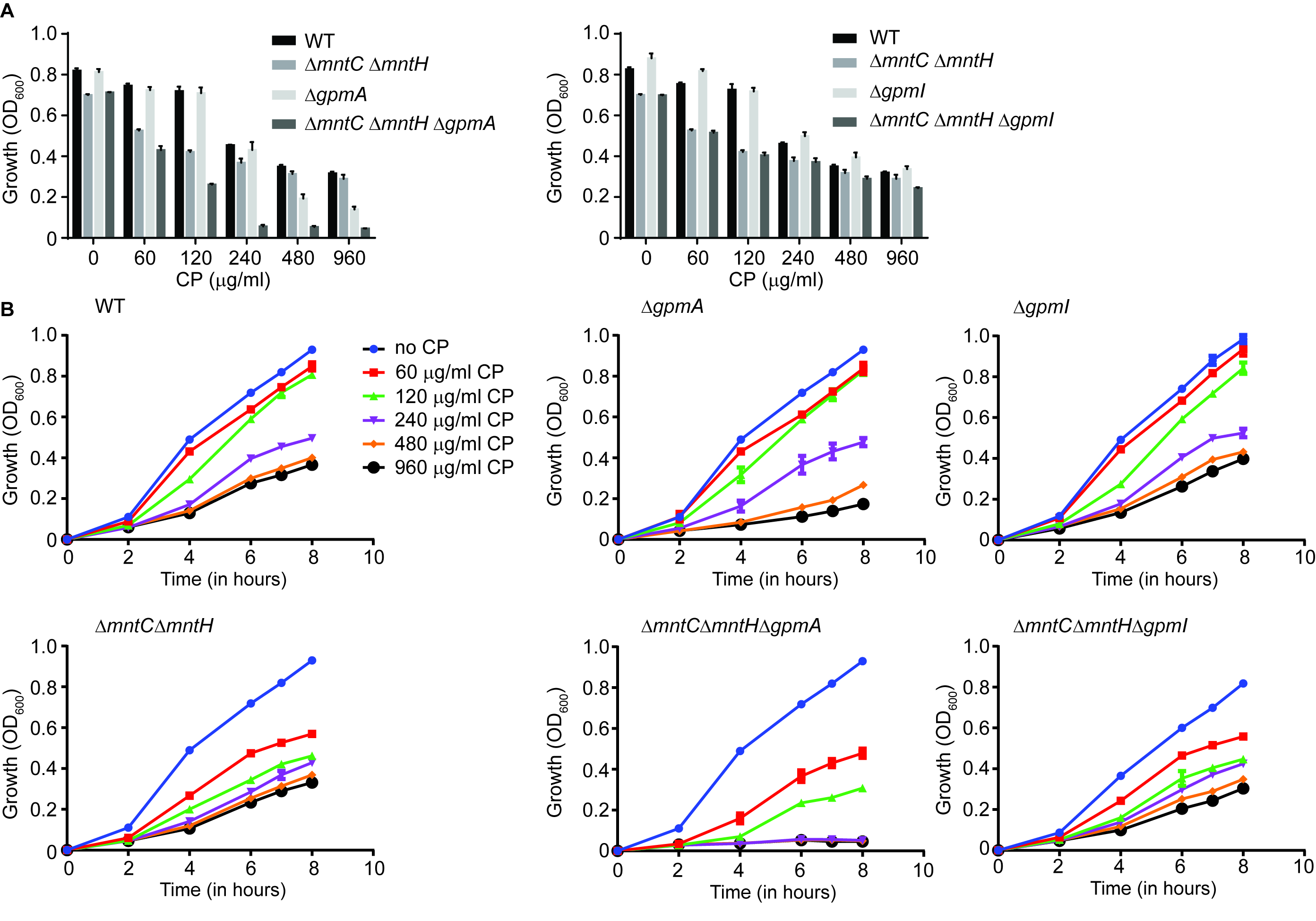

Supplement: S3 Fig — Growth curves for the data presented in Fig 4. (A & B) The growth of wild type, ΔgpmA, ΔmntC ΔmntH and ΔmntC ΔmntH ΔgpmA, ΔgpmI, ΔmntC ΔmntH and ΔmntC ΔmntH ΔgpmI were assessed in rich medium supplemented with 1 μM MnCl2 and 1 μM ZnSO4 in the presence of increasing concentrations of CP. Panel A shows the optical density of wild type mutant strains at t = 8 before normalization to either wild type or the ΔmntC ΔmntH background. (TIF) [file ppat.1007971.s003.tif]

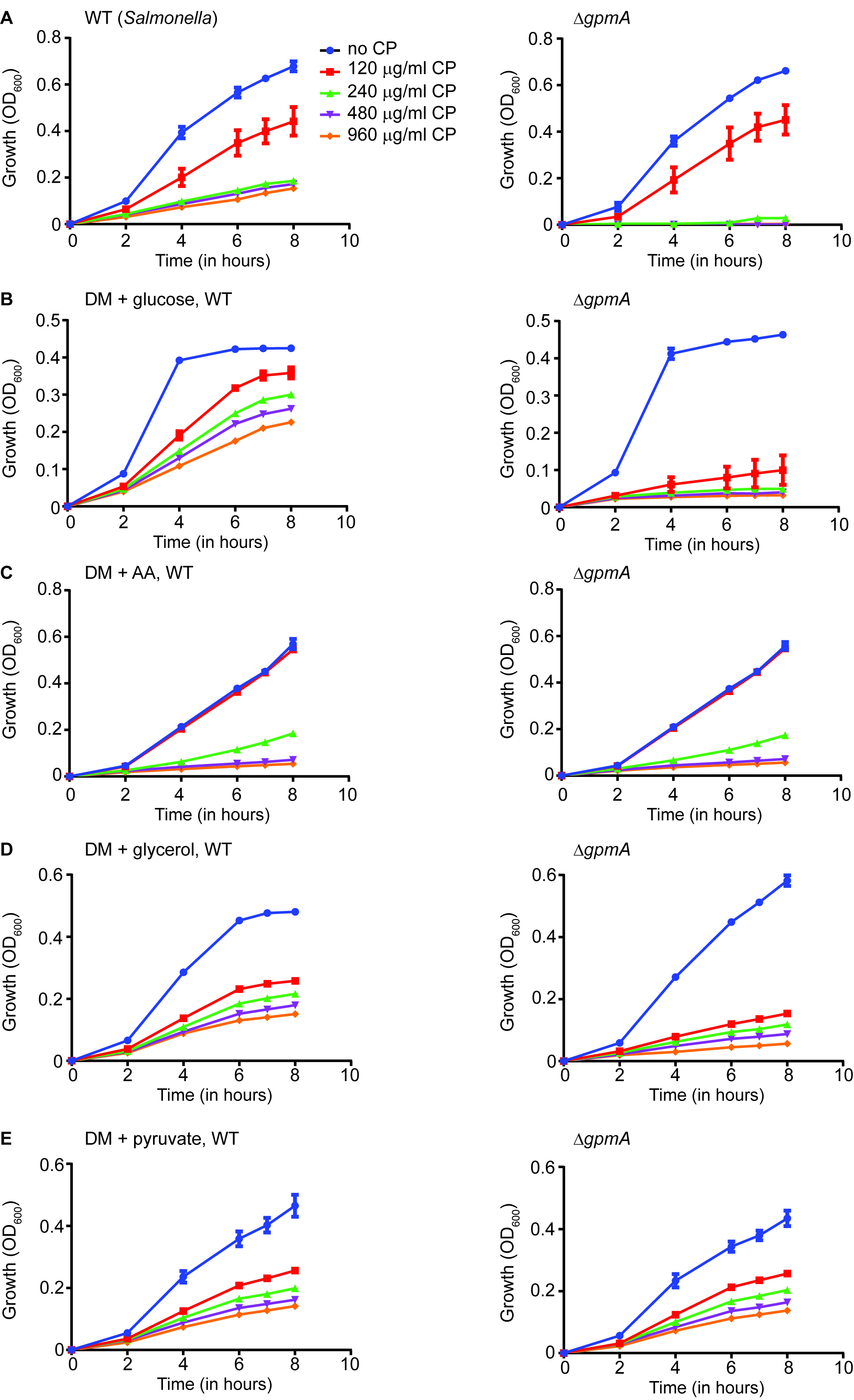

Supplement: S4 Fig — Growth curves for the data presented in Fig 6. (A) Wild type Salmonella and ΔgpmA were grown in rich medium in the presence of increasing concentrations of CP. (B-E) Wild type Salmonella and ΔgpmA were grown in defined medium (DM) supplemented with (B) glucose (DM + glucose), (C) Casamino acids (DM + AA), (D) glycerol (DM + glycerol) or (E) sodium pyruvate (DM + pyruvate) as a carbon source in the presence of increasing concentrations of CP. (TIF) [file ppat.1007971.s004.tif]
